# Prevalence of iron deficiency and iron deficiency anaemia among children with congenital heart defects at tertiary hospitals in Dar es Salaam, Tanzania: a cross-sectional study

**DOI:** 10.11604/pamj.2022.43.175.30944

**Published:** 2022-12-05

**Authors:** Yasser Habresh Said, Evelyne Assenga, Emmanuel Munubhi, Rodrick Kisenge

**Affiliations:** 1 Department of Paediatrics and Child Health, Muhimbili University of Health and Allied Sciences, Dar es Salaam, Tanzania,; 2 Department of Paediatrics and Child Health, Muhimbili National Hospital, Dar es Salaam, Tanzania

**Keywords:** Iron deficiency, iron deficiency anaemia, congenital heart defects, children, Tanzania

## Abstract

**Introduction:**

iron deficiency (ID) is the most prevalent nutritional problem worldwide with children being the most vulnerable. In children with congenital heart defect (CHD), ID may lead to iron deficiency anaemia (IDA) which carries a poor prognosis due to exacerbation of left ventricular dysfunction and heart failure. This study assessed the prevalence and factors associated with ID and IDA among children with CHD at Muhimbili National Hospital (MNH) and Jakaya Kikwete Cardiac Institute (JKCI) in Tanzania.

**Methods:**

a descriptive hospital-based cross-sectional study was conducted among 238 participants with echocardiography confirmed CHD presenting at MNH and JKCI. A structured questionnaire was used to collect demographic data and medical history. Anthropometric measurements were done and blood samples for evaluation of complete blood count, serum ferritin and C-reactive protein were collected. Descriptive statistics such as frequencies, percentages, median with interquartile range, were used to describe study participants. Comparison of continuous variables was performed using Student's t-test or Mann-Whitney U-test as appropriate and Chi-square (x2) test or Fisher's exact test for categorical variables to determine associations. Odds ratio (OR) with 95% confidence intervals (CI) were estimated to determine risk factors for iron deficiency and iron deficiency anaemia. All analyses were conducted using SPSS version 20 and p-value ≤0.05 was considered statistically significant.

**Results:**

characteristic of study participant majority 66.4% (n 158) were less than 60 month of age with nearly equal number of male 51.3%(122) to female 48.7% (n 116). The overall prevalence of anaemia among study participants was 47.5% (n 238) with mild, moderate and severe anaemia being 21.4%, 21.4% and 4.6% respectively. The prevalence of iron deficiency was 26.9% (n 64) and that of iron deficiency anaemia was 20.2% (n 48). Age below 5 years, cyanotic CHD, history of recent illness and less consumption of red meat were significantly associated with iron deficiency (ID) and iron deficiency anaemia (IDA). After controlling for independent variables, history of recent illness aOR 0.46, 95% CI 0.22-0.98 P 0.045 less frequent consumption of red meat aOR 0.11 95% CI 0.04-0.32 P <0.001 and cyanotic CHD aOR: 0.40, 95% CI 0.18-0.87; p 0.021, were associated with of iron deficiency similarly age below 5 years aOR 0.33 0, 95% CI 14-0.89 P 0.02 early weaning practices aOR 0.50 95% CI 0.23-0.97 P 0.050 less frequent consumption of red meat aOR 0.07 CI (0.02-0.24 p <0.01 were significantly associated with iron deficiency anaemia.

**Conclusion:**

nearly half of the children with CHD in this study had anaemia, more than a quarter had ID and one-fifth had IDA. Routine screening and management of both ID and IDA in children with CHD should be practised during weaning and throughout the childhood to prevent ventricular dysfunction further heart failure.

## Introduction

Iron deficiency (ID) is the most prevalent micronutrient deficiency worldwide [1-3] exerting negative effects on a great number of children in developing counties. Iron deficiency accounts for up to 50% of anaemia in children from malaria endemic areas, affecting mostly children under five years of age [4,5].

In Tanzania, the 2010 Demographic Health Survey (DHS) indicated that the prevalence of anaemia among children aged 6-59 months was 59%, of whom 35.3% had iron deficiency and 24.0% had iron deficiency anaemia (IDA) [6]. In 2015-2016, the prevalence of anaemia remained stable at 58% [7]. Risk factors for ID and IDA included: young age, region of residency, socioeconomic status, poor weaning practices, frequent infections and lack of micronutrient supplementation [6].

In children with congenital heart disease (CHD), presence of IDA worsens or exacerbates left ventricular dysfunction and heart failure, leading to an increased blood viscosity due to compensatory increase in red blood cell mass [8] This can be a risk factor for cerebral vascular disease [9,10]. There is paucity of information about the magnitude of ID and IDA in children with CHD in Tanzania. This study assessed the prevalence and factors associated with ID and IDA among children with CHD presenting at Muhimbili National Hospital (MNH) and Jakaya Kikwete cardiac institute (JKCI) in Dar es Salaam, Tanzania.

## Methods

**Study design and setting:** this was a hospital based descriptive cross-sectional study conducted at Muhimbili National Hospital (MNH) and Jakaya Kikwete Cardiac Institute (JKCI) in Dar es Salaam. Both MNH and JKCI serve as national referral centres receiving paediatric cardiac patients from all regions in Tanzania. Overall, these two health facilities attend about 1500 outpatients and have about 200 admissions per day.

**Study population:** children below 18 years of age with echocardiography confirmed diagnosis of CHD, who presented at MNH and JKCI and whose parents consented were recruited in this study. Sample size calculated were 238 based on prevalence of iron deficiency in Kenya [11]. We excluded children who had received iron supplementation for more than one month, had received blood transfusion within 3 months prior to the study, who were known to have sickle cell disease, or who had undergone corrective cardiac surgery.

**Data collection:** demographic characteristics socioeconomic and of the parents were collected using structured questionnaire data collected were: Age, marital status, education, occupation and level of income. Information pertaining to the child's age, birth weight, breastfeeding and time of weaning were also collected. The type of CHD was also documented from the child's medical notes and their anthropometric measurements were taken and analysed using the WHO growth charts. We collected two venous blood samples of 2 millilitres each in plain and EDTA-anticoagulant tubes using a 5 millilitre syringe for assessment of complete blood count, serum ferritin and C-reactive protein (CRP). Blood sample collection followed the best practices in phlebotomy recommended by WHO [12]. The blood samples collected were labelled with appropriate participants' identification number and immediately taken to the Muhimbili National Hospital Central Pathology Laboratory for analysis. Complete blood count was performed using *Abbott Cell-Dyn 3700 Haematology analyser*, (Abbott Diagnostics) an automated analyser that generates haematologic measurements including: white blood cells, red blood cells, haemoglobin concentration, MCV and MCH from EDTA-anticoagulated whole blood. Analyses of ferritin and CRP were performed using *Abbott Architect Plus ci4100 chemistry analyser* (Abbott Diagnostics).

### Definition of terms

***Anemia:*** is defined based on hemoglobin concentration of <11.0g/dL (for children <5 years), <11.5g/dL (for children aged 5-11 years) and <12.0g/dL (for children aged 12-14 years) using the WHO classification.

***Iron deficiency (ID):*** is a depletion of body's iron stores as indicated by serum ferritin (SF) level below lower limit for age (SF<12.0µg/L for children ≤5 years and SF<15.0µ/L for children 5 years and above. In the presence of infection/inflammation (indicated by raised CRP), iron deficiency is defined as serum ferritin level <30.0µg/L for children ≤5 years and <50.0 µg/L for children >5 years.

***Iron deficiency anaemia (IDA):*** this is defined using hemoglobin level based on child's age and presence or absence of infection/inflammation. For children <5 years, iron deficiency anemia is defined as low haemoglobin level of <11.0g/dL and depleted body iron stores as indicated by SF<12.0µg/L (in absence of infection/inflammation) or SF<30.0µg/L (in presence of infection/inflammation indicated by raised CRP). For children >5years, iron deficiency anemia is defined as low haemoglobin level of <11.5g/dL and depleted body iron stores as indicated by SF<15.0µg/L (in absence of infection/inflammation) or SF<50.0µg/L (in presence of infection/inflammation indicated by raised CRP).

**Statistical analysis:** data were analysed using SPSS version 20. Descriptive statistics including frequencies, percentages, median with interquartile range, were used to describe the study participants. Univariate analysis was done for continuous variables using Student's t-test or Mann-Whitney U-test as appropriate. Categorical variables were compared using the Chi-square (x2) test or Fisher's exact test to determine associations between dependent and independent variables. The odds ratio (OR) with corresponding 95% confidence intervals were estimated to determine risk factors independently associated with iron deficiency and iron deficiency anaemia among children with CHD. Only variables that attained p-value<0.2 during bivariable analysis were included in the multivariable analysis and p-value ≤0.05 was considered statistically significant.

**Ethical considerations:** ethical approval was obtained from the Ethics Review Committee of the Muhimbili University of Health and Allied Sciences. Permission to conduct the study was also sought from participating institutions. A written informed consent was obtained from the parent for all children prior to enrolment. In addition to parental consent, an assent was obtained for children aged 7 years and above. Participation was voluntary and confidentiality of collected information was maintained by the use of a unique study participant identification number.

## Results

**General characteristics of the study population:** a total of 238 children aged between 2 weeks and 13.5 years were included in the final analysis. The median age was 36 months with an (interquartile range) of (17-72) months. Two-thirds (66.4%) of the participants were under 5 years of age. Sex distribution was almost similar with boys consisting of 51.3%. The mean birth weight was 2.80±0.55 kilograms with 21.1% having had low birth weight. Majority (81.9%) of the participants were born at term. Most of the mothers were aged between 25 and 34 years (58.8%), whilst majority were married (87.4%) and had either primary or secondary education level (80.2%) (Table 1). Majority of the participants (80.97%) had acyanotic CHD with VSD being the most common presenting type (30.7%) followed by PDA which was found in 23.5% of all participants (Table 2).

**Table 1:** characteristics of the study participants

Parameter	Frequency (N=238)	Percentage (%)
**Child age**		
<60 months	158	66.4
≥60 months	80	33.6
**Child sex**		
Male	122	51.3
Female	116	48.7
**Birth weight**		
Underweight	50	21.1
Normal weight	187	78.9
**Gestational age at birth**		
Term delivery	195	81.9
Preterm delivery	43	18.1
Age of mother*		
<25 years	7	2.9
25-34 years	140	58.8
≥35 years	89	37.4
Marital status of mother*		
Never married	12	5.0
Married	208	87.4
Divorced/separated	15	6.3
Widow	1	0.4
Education level of mother*		
No formal education	16	6.7
Primary education	111	46.6
Secondary education	80	33.6
University/College	29	12.2
Occupation of mother*		
Housewife	115	48.3
Employed	37	15.5
Self-employed	49	20.6
Peasant	34	14.3
Other	1	0.4

**Table 2:** types of presenting CHD among study participants

Parameter	Frequency (N=238)	Percentage (%)
**Type of CHD**		
Cyanotic CHD	46	19.3
Acyanotic CHD	192	80.7
**CHD condition**		
VSD	73	30.7
ASD	25	10.5
PDA	56	23.5
TOF	39	16.4
VSD and PDA	8	3.4
PS	9	3.8
Other types	28	11.8

**Prevalence of iron deficiency and iron deficiency anemia:** the prevalence of anaemia was 47.5% while that of iron deficiency and iron deficiency anaemia was 26.9% and 20.2% respectively. Non-iron deficiency anaemia accounted for the remaining 27.3% and this did not differ significantly between age groups (Table 3).

**Table 3:** prevalence of ID and IDA among study participants

Parameter	Frequency (N=238)	Percentage (%)
**Anaemia status**		
Absent	125	52.5
Present	113	47.5
**Iron status**		
Normal	174	71.3
Depleted	64	26.9
**Iron deficiency anaemia**		
Absent	190	79.8
Present	48	20.2
**Non-iron deficiency anaemia**		
Absent	173	72.7
Present	65	27.3

**Factors associated with iron deficiency and iron deficiency anemia:** children aged five years and above had a 54% reduced risk for ID (COR=0.46, 95% CI: 0.24-0.89) compared to those below 5 years. Having acyanotic CHD was associated with a 51% decreased risk for iron deficiency compared to cyanotic CHD (COR=0.49, 95% CI: 0.25-0.97). Children who had been well (COR=0.39, 95% CI: 0.20-0.76), and those who consumed red meat at least twice a week also had a significantly decreased risk of iron deficiency (COR=0.15, 95% CI: 0.06-0.36) (Table 4). Children aged five years and above, having no recent illness, consuming red meat at least twice a week was significantly associated with a decreased risk of iron deficiency anaemia (Table 5).

**Table 4:** univariable and multivariable analysis of factors associated with of ID among children with CHD

Parameter	N	Crude OR (95% CI)	P-value	Adjusted OR (95% CI)	P-value
**Age**					
<60 months	158	Ref		Ref	
≥60 months	80	0.46 (0.24-0.89)	**0.022**	0.87 (0.40-1.90)	0.723
**Sex**					
Male	122	Ref			
Female	116	0.70 (0.39-1.24)	0.221	-	-
**Birth weight categories**					
Underweight	50	Ref			
Normal weight	187	1.07 (0.52-2.17)	0.857	-	-
**Gestational age at birth**					
Term delivery	195	Ref			
Preterm delivery	43	0.67 (0.30-1.50)	0.332	-	-
**Weaning practices**					
At < 6 months	125	Ref		Ref	
At ≥ 6 months	98	0.60 (0.33-1.11)	0.104	0.64 (0.33-1.26)	0.198
Still on EBF	8	0.32 (0.04-2.65)	0.288	0.08 (0.02-1.69)	0.999
**Type of CHD**					
Cyanotic CHD	46	Ref		Ref	
Acyanotic CHD	192	0.49 (0.25-0.97)	**0.039**	0.40 (0.18-0.87)	**0.021**
**Child recent sick**					
Yes	49	Ref		Ref	
No	189	0.39 (0.20-0.76)	**0.006**	0.46 (0.22-0.98)	**0.045**
**Consumption of red meat**					
Never	43	Ref		Ref	
Once per week	99	0.66 (0.32-1.37)	0.266	0.48 (0.20-1.15)	0.099
Twice/more per week	95	0.15 (0.06-0.36)	**<0.001**	0.11 (0.04-0.32)	**<0.001**
**Child wasting**					
Normal weight	106	Ref			
Malnourished	104	1.25 (0.69-2.2)	0.467	-	-
**Child stunting**					
Normal	162	Ref			
Stunted	59	1.13 (0.62-2.07)	0.694	-	-

**Table 5:** univariable and multivariable analysis of factors associated with of IDA among children with CHD

Parameter	N	Unadjusted OR (95% CI)	P-value	Adjusted OR (95% CI)	P-value
**Age**					
<60 months	128	Ref		Ref	
≥60 months	80	0.27 (0.12-0.64)	**0.003**	0.33 (0.14-0.89)	**0.025**
**Sex**					
Male	122	Ref			
Female	116	0.86 (0.46-1.63)	0.652	-	-
**Birth weight categories**					
Underweight	50	Ref			
Normal weight	187	0.88 (0.41-1.87)	0.729	-	-
**Gestational age at birth**					
Term delivery	195	Ref			
Preterm delivery	43	0.94 (0.42-2.13)	0.890	-	-
**Weaning practices**					
At < 6 months	125	Ref		Ref	
At ≥ 6 months	98	0.50 (0.25-1.01)	0.055	0.50 (0.23-0.97)	**0.050**
Still on EBF	8	0.43 (0.05-3.66)	0.442	0.09 (0.00-1.82)	0.999
**Type of CHD**					
Cyanotic CHD	46	Ref			
Acyanotic CHD	192	1.51 (0.63-3.63)	0.354	-	-
**Child recent sick**					
Yes	49	Ref		Ref	
No	189	0.48 (0.24-0.98)	**0.044**	0.55 (0.24-1.29)	0.168
**Consumption of red meat**					
Never	43	Ref		Ref	
Once per week	99	0.47 (0.22-1.00)	0.050	0.42 (0.17-1.02)	0.056
Twice/more per week	95	0.06 (0.02-0.20)	**<0.01**	0.07 (0.02-0.24)	**<0.01**
**Child wasting**					
Normal weight	106	Ref			
Wasted	104	1.69 (0.84-3.41)	0.142	-	-
**Child stunting**					
Normal	162	Ref			
Stunted	59	1.08 (0.50-2.37)	0.839	-	-

After controlling for all independent variables, having acyanotic CHD (AOR=0.40, 95% CI: 0.18-0.87), being well (AOR=0.46, 95% CI: 0.22-0.98), and consumption of red meat at least twice a week (AOR=0.11, 95% CI: 0.04-0.32) were significantly protective against iron deficiency (Table 4). In multivariable adjusted analysis, the factors which remained protective against iron deficiency anaemia were age 5 years and above (AOR=0.33, 95% CI: 014-0.89), and exclusive breastfeeding for 6 months (AOR=0.50, 95% CI: 0.23-0.97). Consumption of red meat at least twice per week also carried a protective effect against iron deficiency anaemia (AOR=0.07, 95% CI: 0.02-0.24) (Table 5).

## Discussion

This study assessed the prevalence anaemia and factors associated with iron deficiency and iron deficiency anaemia among children with CHD attending tertiary referral hospitals in Tanzania. The most common CHD were VSD. The prevalence of ID and IDA were 26.9% and 20.2% respectively were by age below 5 years, history of recent illness, and less frequent consumption of red meat were significantly associated with ID and IDA among children with CHD. Presence of cyanotic CHD was a risk factor for iron deficiency, while early weaning was associated with iron deficiency anaemia.

The prevalence of children with anaemia found in this study is lower than that reported among Iranian paediatric patients with acyanotic and cyanotic CHD [13]. Higher proportion of anaemia in the Iranian study can partly be explained by the fact that, in their study they used higher cut-offs for the diagnosis of anaemia than the one we used in our study. For example, in their study they used haemoglobin cut-offs of <12g/dl for acyanotic patients and <15g/dl for cyanotic patients while, in our study we used age specific cut-offs for the diagnosis of anaemia according to the WHO [14], nevertheless, the proportion of anaemia found in our study was lower than the proportion reported from the Tanzanian demographic and health survey [15], possibly because this study focused on children with CHD rather than the general paediatric population as in the demographic survey. Similarly, the proportion is lower than that reported in a hospital based survey conducted in Mwanza [16] and a school based survey conducted in Tanga Region [17]. While this study included both children under 5 years and above 5 years, the study in Mwanza included children aged 6-59 months and that in Tanga included children aged 7-14 years. The proportion of iron deficiency in this study is higher than the proportion of iron deficiency which was reported in Kenya and India among children with cyanotic CHD [11,18].

In contrast to the Kenyan and Indian studies, this study included children with both cyanotic and acyanotic CHD. While we found 26.9% of participants had iron deficiency, a study in Albania which analysed blood samples of 114 children with CHD found that 85.9% had iron deficiency requiring iron supplementation [19]. The high proportion of iron deficiency in the Albanian study might be attributed to the fact that the study involved inpatient children only who are likely to have other co morbidities which may further lead to ID; unlike our study which included outpatients.

In comparison to other studies from Tanzania, the proportion of iron deficiency found in our study is different to that from two previous studies in other parts in Tanzania one reporting a lower proportion of iron deficiency of 22.6% [16] and the other reporting a slightly higher proportion (32.7%) of iron deficiency among school children [17]. Moreover, the proportion of iron deficiency found in our study is lower than that reported in a study in Abia State, Nigeria [20]. These differences can partly be explained by the differences in the setting and the population that were studied and the fact that in our study we recruited children coming from both rural and urban communities.

In this study, we have demonstrated that one in five children with CHD has IDA. In 1993, Gaiha *et al*. reported a lower prevalence of IDA of 18.2% among cyanotic CHD patients. However, this lower prevalence was attributed to the study population which comprised of adolescents and young adults whose risk is much lower [18]. In another study conducted by Olcay *et al*. the prevalence of IDA was found to be 52.2% among children aged 1-9 years with CHD [21]. A more recent study from India has found a prevalence of IDA of 47.06% among children with cyanotic CHD aged ≤12 years. High prevalence of IDA in the Indian study is again attributable to the study population which comprised of a large proportion (>60%) of infants and toddlers in whom nutritional ID may have been co-existent [13].

This study has demonstrated that factors significantly associated with ID and IDA were age below 5 years, history of recent illness and less frequent consumption of red meat. Presence of cyanotic CHD was a risk factor for iron deficiency, while early weaning was associated with iron deficiency anaemia. Iron deficiency and iron deficiency anaemia were significantly higher among children under the age of five years compared to those above five years. This finding is not uncommon. In the study conducted in Kenya, all children who had iron deficiency were below 5 years of age [11]. Iron deficiency anaemia is the most common cause of anaemia in younger children. The risk for anaemia is increased after the age of 6 months when the child is introduced to complementary foods and is the age at which they are more exposed to contaminants [22], and consequently increased risk of infectious morbidity.

Poor nutritional practices including early child weaning have been strongly associated with child malnutrition, and micronutrient deficiencies. Both early introduction of complementary foods [23] and exclusive breastfeeding beyond 6 months have been reported to be associated with increased risk of anaemia [24,25]. In this study, optimal exclusive breastfeeding and weaning at 6 months was associated with significant reduction in the risk of iron deficiency anaemia.

This study found that a history of recent illness in the child (within the past one month) was associated with iron deficiency, but not iron deficiency anaemia. Tanzania is a malaria endemic country, and a previous study reported high malaria parasitaemia to be associated with anaemia [16]. In our study, 20.6% of the participants reported a history of recent illness. Although we did not inquire information about specific diagnoses for the child's recent illness, fever, cough and difficulty in breathing were the most reported complaints.

This study found that consumption of red meat at least twice a week to be associated with significant decreased risk of anaemia, iron deficiency and iron deficiency anaemia. These findings concur with those of a study conducted in Israel in which children who had extremely low red meat consumption had a 4-fold increased rate of iron deficiency [26]. Finally, this study also found significant association between presence of cyanotic CHD with the risk of iron deficiency. Overall, cyanotic CHD have been associated with iron deficiency and iron deficiency anaemia more than acyanotic CHD [27]. This is explained by the physiological response of increased erythropoietin (EPO) which leads to a rapid increase in red cell production in a backdrop of similar nutritional status, hence leading to iron deficiency due to increased demand. Given that the participants were from nearly all regions in Tanzania, this study provides a broader picture of iron deficiency and iron deficiency anaemia in the paediatric population of CHD patients in Tanzania. The cross-sectional study design does not allow drawing of causal associations between independent variables with iron deficiency and iron deficiency anaemia. Moreover, we did not recruit a comparison group of children without CHD, which could have strengthened observations and drawing of conclusions.

## Conclusion

The overall proportion of anaemia, iron deficiency and iron deficiency anaemia among children with CHD in Tanzania was found to be high. The risk factors for iron deficiency and iron deficiency anaemia were; age below 5 years, history of recent illness and less frequent consumption of red meat Presence of cyanotic CHD was a risk factor for ID, while early weaning was associated with IDA. Children with CHD, especially those aged below 5 years, should be screened for iron deficiency and iron deficiency anaemia and managed accordingly.

### What is known about this topic

- *Iron deficiency and iron deficiency anemia are preventable in children;*
- *Iron deficiency carries poor prognosis in children with congenital heart disease;*
- *Iron deficiency anemia increased risk of heart failure*.

### What this study adds

- *Cyanotic congenital heart disease carries higher risk for iron deficiency and iron deficiency anemia*.
